# Supplementary material for: Control Over the Morphology of Electrospun Microfibrous Mats of a Polymer of Intrinsic Microporosity
Source: Membranes (Basel). 2021 May 31;11(6):422. doi: 10.3390/membranes11060422 (PMC8227142; doi:10.3390/membranes11060422)
Supplement: Supplementary file 1 [file membranes-11-00422-s001.zip › membranes-1205808-supplementary.pdf]

membranes

MDPI

Supplementary Materials

Control Over the Morphology of Electrospun Microfibrous Mats of a Polymer of Intrinsic Microporosity

Elsa Lasseuguette [1,*], Richard Malpass-Evans [2], John M. Tobin [2], Neil B. McKeown [2] and Maria-Chiara Ferrari [1]

[1] School of Engineering, University of Edinburgh, Robert Stevenson Road, Edinburgh EH9 3FB, UK; M.Ferrari@ed.ac.uk
[2] EaStCHEM School of Chemistry, University of Edinburgh, David Brewster Road, Edinburgh EH9 3FJ, UK; R.Malpass-Evans@ed.ac.uk (R.M.-E.); J.Tobin@ed.ac.uk (J.M.T.); Neil.McKeown@ed.ac.uk (N.B.M.)
* Correspondence: e.lasseuguette@ed.ac.uk

Citation: Lasseuguette, E.; Malpass-Evans, R.; Tobin, J.M.; McKeown, N.B.; Ferrari, M.-C. Control over the morphology of electrospun microfibrous mats of a polymer of intrinsic microporosity. *Membranes* **2021**, *11*, 422. https://doi.org/10.3390/membranes11060422

Academic Editor: Rasel Das and Guorong Xu

Received: 16 April 2021
Accepted: 27 May 2021
Published: 31 May 2021

Publisher's Note: MDPI stays neutral with regard to jurisdictional claims in published maps and institutional affiliations.

Copyright: © 2021 by the authors. Submitted for possible open access publication under the terms and conditions of the Creative Commons Attribution (CC BY) license (https://creativecommons.org/licenses/by/4.0/).

Physical properties

Table S1. Surface tension of solvent systems.

	Surface tension (mN/m) [+/-0.1 mN/m]
CHCl3/nPL (10/0)	28.31
CHCl3/nPL (9/1)	28.32
CHCl3/nPL (7/3)	28.43
CHCl3/nPL (5/5)	28.83
CHCl3/nPL (3/7)	28.93
CHCl3/nPL (1/9)	29.28
CHCl3/nPL (0/10)	29.55

Table S2. Characterizations of electrospinning solutions and corresponding electrospun mats.

	Electrospinning solutions		Electrospun sample				
20%PIMEATB in	Conductivity (µS/cm) [+/- 5%]	Viscosity (Visual aspect)	Contact angle (°)	FD (µm)	PD (µm)	P_{H2O} (10^{-2}Bar) [+/- 0.1]	R_{Air} (10^{+7} m^{-1}) [+/-5%]
CHCl3/nPL (10/0)	0.02	-	126	7.9 (+/-0.5)	51.4 (+/- 0.5)	0.5	4.9
CHCl3/nPL (9/1)	0.08	↓		7.2 (+/- 0.9)	30.3 (+/- 0.5)	2.6	9.2
CHCl3/nPL (7/3)	0.25			5.3 (+/- 0.5)	28 (+/- 0.3)	1	9.5
CHCl3/nPL (5/5)	0.42			4.7 (+/- 0.5)	20.3 (+/- 0.2)	1.3	10.1
CHCl3/nPL (3/7)	0.64		No fibres				
CHCl3/nPL (1/9)	0.78						
CHCl3/nPL (0/10)	0.96	+					

Water contact angle

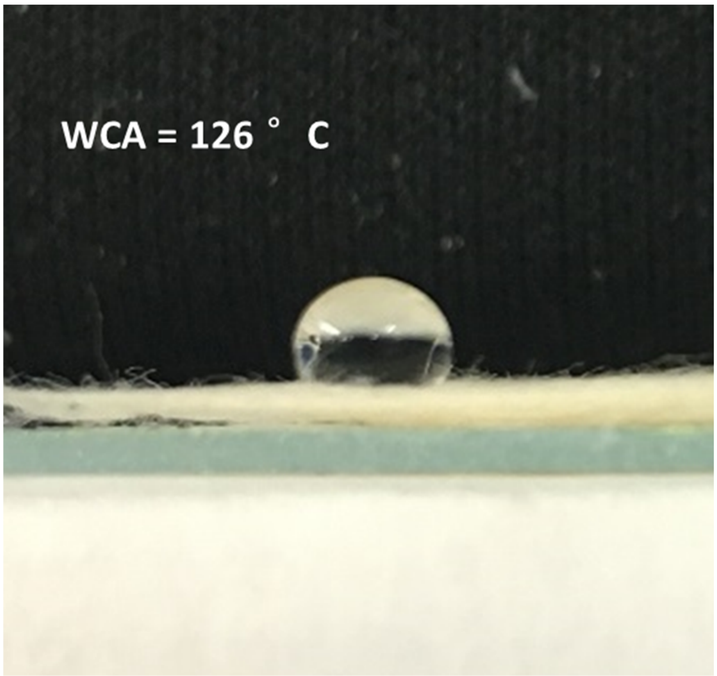

Figure S1: Water contact angle on PIMEATB fibres from solution (9/1).
